# Direct Observations of Office Disciplinary Referrals for Aggressive and Other Disruptive Behaviors in Elementary Schools

**DOI:** 10.3390/bs16091603

**Published:** 2026-09-09

**Authors:** Daniel A. Waschbusch, Pevitr S. Bansal, Marcela C. Ramos, William E. Pelham, Greta M. Massetti

**Affiliations:** 1 Department of Psychiatry and Behavioral Health, Penn State Health, Penn State College of Medicine, 500 University Drive, Hershey, PA 17033, USA; 2 Department of Psychology, Montclair State University, 1 Normal Avenue, Montclair, NJ 07043, USA; bansalp@montclair.edu; 3 Center for Children and Families, Department of Psychology, Florida International University, Miami, FL 33199, USA; marcramo@fiu.edu (M.C.R.);; 4 Department of Public Health Sciences, Georgia State University, 140 Decatur Street SE, Atlanta, GA 30303, USA; gmassetti@gsu.edu

**Keywords:** aggression, office discipline referrals, elementary school, school discipline, direct observation, instructional time, school-based intervention

## Abstract

Aggressive behavior in childhood is a major developmental and educational concern, but school discipline records provide limited information about how aggression is identified and managed in everyday settings. This study used direct observation to characterize office discipline referral (ODR) events among kindergarten through fifth-grade students in 2007, with each of 14 schools observed for one designated week near the end of a randomized school-based intervention trial. Observers recorded 348 referrals involving 296 students. Aggressive or destructive behavior—physical aggression, verbal aggression, or property destruction—accounted for 46.8% of referrals, with physical aggression alone accounting for 38.5%. Aggressive/destructive referrals involved descriptively longer office stays than other referrals, although the within-school difference was smaller and imprecisely estimated after accounting for school-level clustering. Fifth grade had the highest overall referral rate, whereas kindergarten referrals were proportionally most likely to involve aggressive/destructive behavior. Exploratory matched-pair comparisons provided little evidence of differences between intervention and comparison schools in aggressive/destructive referral rates, although some differences in referral composition and prolonged office stays remained imprecisely estimated. ODRs capture a consequential but selective subset of childhood aggression: behavior that is observed, interpreted, and formalized through school disciplinary procedures.

## 1. Introduction

Aggressive behavior problems in childhood are a major developmental, educational, and public health concern. They are associated with academic underachievement, impaired peer and adult relationships, and elevated risk for later psychosocial difficulties (Tremblay et al., 2018). Aggression is also heterogeneous. In elementary schools, it may include physical aggression, verbal aggression, threats, intimidation, property destruction, or other severe disruptive behavior that compromises classroom functioning or peer safety. These behaviors differ in form, severity, developmental meaning, likely function, and the degree to which they require immediate adult intervention. Understanding aggression in school settings therefore requires attention not only to children’s behavior but also to how that behavior is observed, interpreted, and managed.

Schools provide a particularly important context for studying these processes because children interact with peers and nonfamilial adults under structured behavioral expectations (Waschbusch et al., 2019). Teachers and other school personnel observe behavior across classrooms, hallways, playgrounds, cafeterias, and other settings, giving them access to behavior that may not be visible to parents or clinicians. Schools also make consequential decisions in response to aggression, including whether behavior can be managed in the classroom, whether administrative involvement is needed, and whether a student is temporarily or permanently removed from instruction. School responses therefore represent more than administrative outcomes; they are part of the context in which aggressive behavior is interpreted and managed (Skiba et al., 2014).

Office discipline referrals (ODRs) are one of the most common ways schools document aggressive and disruptive behavior judged to exceed classroom-management thresholds. ODRs are routinely collected and are often used to monitor school-wide behavior, identify students who may need additional support, allocate intervention resources, and evaluate prevention efforts (Irvin et al., 2004, 2006; McIntosh et al., 2010; Sugai et al., 2000). For aggression research, their value lies in capturing behavior that has become institutionally consequential; an incident was observed, interpreted as sufficiently serious to warrant formal action, and entered into the school disciplinary system. At the same time, this process means ODRs are not direct or complete measures of childhood aggression. A referral reflects both the student behavior that occurred and the sequence of adult judgments and school procedures that transformed that behavior into a formal disciplinary event (McIntosh et al., 2009, 2011; Pas et al., 2011). Consequently, differences in ODRs may reflect differences in student behavior, adult response thresholds, classroom management practices, school discipline policies, administrative procedures, or some combination of these factors (Bradshaw et al., 2010; Girvan et al., 2017; Wright & Dusek, 1998).

We use the term school-managed aggression to refer to aggressive or destructive behavior that school personnel identify as requiring formal administrative involvement. This is a narrower construct than aggression occurring in school more generally. Some aggressive behavior may not be observed by adults, may be resolved informally, may be managed within the classroom, or may be interpreted differently depending on the adult, classroom, school, or student involved (Martella et al., 2010; Nelson et al., 2002; Pas et al., 2011). School-managed aggression therefore captures the subset of aggressive behavior that crosses a referral threshold and enters the formal discipline process. This subset is important because it identifies incidents that have become consequential for the student, peers, classroom, and school.

Distinguishing school-managed aggression from other office-referred behavior is also important because aggressive and disruptive behaviors are heterogeneous. Physical aggression, verbal aggression, property destruction, disruption, and noncompliance differ in severity, likely function, safety implications, and the responses they may evoke from adults (Fite et al., 2017; Kaufman et al., 2010; Loeber & Schmaling, 1985; Loeber et al., 1993). Behaviors such as physical aggression or property destruction may create immediate safety concerns and prompt rapid administrative involvement, whereas disruption, insubordination, or disrespect may depend more heavily on adult interpretation, classroom norms, and referral thresholds. Comparing aggression-related referrals with other referrals can therefore help clarify whether aggressive incidents differ not only in the behavior that precipitates referral, but also in the disciplinary response that follows.

Despite their practical value, standard administrative ODR records often provide limited information about the process surrounding a referral. Even when a referral reason is recorded, administrative data may not indicate precisely what behavior occurred, what classroom strategies were attempted before referral, how long the student remained outside instruction, or what occurred before the student returned to class (McIntosh et al., 2011; Pas et al., 2011; Wright & Dusek, 1998). These omissions limit researchers’ ability to determine how aggressive and disruptive behavior is translated into disciplinary consequences and limit schools’ ability to evaluate whether their referral procedures are functioning as intended.

Time removed from instruction is one particularly important but undermeasured consequence of office referral. Each ODR may involve some interruption of classroom participation, but the duration and nature of that interruption can vary substantially. A brief office visit may allow de-escalation, problem-solving, caregiver contact, or administrative follow-up, whereas an extended office stay may more closely resemble exclusion from instruction, with potential consequences for academic engagement and classroom participation (Noltemeyer et al., 2015; Skiba et al., 2014). Thus, two incidents that each count as one ODR may differ markedly in their educational consequences. Measuring the duration of office removal can therefore add information that referral counts alone cannot provide.

Direct observation of referral events provides one way to examine these otherwise hidden features of the discipline process. Although intensive observation is unlikely to be practical for routine school monitoring, it can capture information that administrative records often omit, including the specific behavior associated with referral, the amount of time spent in the office, and the classroom strategies teachers report using beforehand. For the study of school-managed aggression, these data make it possible to examine both sides of the referral event: the aggressive or disruptive behavior that prompted formal action and the school response that followed. Direct observation can therefore help clarify not simply how often aggression reaches the office-referral threshold, but what happens once it does.

The present study used direct observational data collected during a randomized trial of the Academic and Behavioral Competencies (ABC) school intervention model. ABC was one component of the multisite Social and Character Development program (SACD; Social and Character Development Research Consortium, 2010) and included school-wide behavioral expectations, systematic monitoring of rule violations, positive daily notes and weekly rewards for students with few rule violations, and more intensive supports for students with persistent behavioral difficulties (Pelham et al., 2005; Waschbusch et al., 2005). The current report focuses on office referral events observed during one designated week per school between early May and early June 2007, near the end of the third intervention year. Because comparable baseline observational data were not available, the intervention–comparison analyses cannot determine whether any observed post-test differences were caused by ABC. Accordingly, the primary purpose of the present study was descriptive: to characterize office-referral events, particularly those involving school-managed aggression. Condition comparisons were retained as exploratory analyses because the schools had originally been randomly assigned within matched pairs and because these contrasts could generate hypotheses about how school-wide behavioral intervention might influence the frequency, composition, or management of office referrals.

The study addressed five questions. First, how frequently were kindergarten-through-fifth-grade students referred to the office during the observation week, and how did referral rates vary across grade levels? Second, what proportion of referrals involved school-managed aggression, defined as physical aggression, verbal aggression, or property destruction? Third, did referrals involving school-managed aggression differ from other referrals in the amount of time students spent in the office? Fourth, what strategies did teachers report using before referral? Fifth, as an exploratory question, how did referral patterns differ between schools assigned to ABC and schools assigned to services as usual? By examining the behavior that prompted referral together with pre-referral teacher responses and the duration of office removal, the study sought to provide a more detailed account of how aggressive and disruptive behavior enters and is managed within elementary-school discipline systems.

## 2. Materials and Methods

### 2.1. Participants

Data were collected from 14 schools participating at one site of the Social and Character Development (SACD) study, a three-year randomized trial of school-based programs designed to reduce problem behavior and promote social and character development among elementary-school students (Haegrich & Metz, 2009; Social and Character Development Research Consortium, 2010). The present analyses were limited to office discipline referrals involving students in kindergarten through fifth grade.

The 14 schools included seven assigned to the ABC intervention and seven assigned to the comparison condition. Ten were public schools in a large urban school district, two were charter schools in the same district, and two were in a nearby suburban district. Across the 14 schools, 6010 students were enrolled in kindergarten through fifth grade during the observation period; school-level K–5 enrollment ranged from 204 to 775 students. School demographic characteristics by condition are presented in Table 1. Many of the schools served economically disadvantaged populations, although socioeconomic composition varied considerably across schools, as reflected in the school-level percentages of students receiving free or reduced-price lunch (see Table 1).

Schools were matched before randomization on enrollment, percentage of students receiving free or reduced-price lunch, and percentage of students identified as English Language Learners. Within each matched pair, one school was randomly assigned to ABC and the other to the comparison condition. As shown in Table 1, the intervention and comparison schools were similar in enrollment and the demographic characteristics available for the present analyses.

### 2.2. Procedures

During the first two years of the intervention, administrative office discipline referral records were available from only 8 of the 14 schools, and several of those schools did not maintain comparable referral records for students in the earlier elementary grades. Because existing administrative records were incomplete and inconsistent across schools, the research team developed a standardized direct-observation procedure to characterize office referral events across all participating schools.

Each school was observed for one designated school week between 7 May and 8 June 2007, near the end of the third intervention year. The intensive one-week protocol was developed to obtain standardized, comparable data across schools after available administrative referral records proved incomplete and inconsistent. The protocol required full-day observer coverage and was implemented for one week at each school. One trained research assistant was assigned to each school during its observation week. Research assistants remained in the school administrative office from before the school day began until after it ended and recorded each disciplinary referral involving a student in kindergarten through fifth grade. A standardized form was used to document information about the student, the reason for referral, and the times the student entered and left the office.

At the end of each school day, research assistants contacted teachers who had referred students to obtain additional information about the events preceding referral. Teachers were asked what strategies they had used before sending the student to the office and approximately how much time they had spent attempting to manage the behavior before referral. These teacher reports supplemented the direct observations of what occurred after the student entered the office.

### 2.3. Measures

#### 2.3.1. Office Discipline Referral Event

An office discipline referral event was defined as an instance in which a kindergarten-through-fifth-grade student was sent, brought, or otherwise referred to the administrative office for disciplinary reasons during the school day. Referrals for non-disciplinary reasons were not included.

#### 2.3.2. Student Information

For each referral, research assistants recorded the student’s name, grade, sex, and school. Identifying information was replaced with study codes after data collection.

#### 2.3.3. Time in Office

Research assistants recorded the times at which each student entered and left the administrative office. These times were used to calculate the total number of minutes the student spent in the office following referral. For analyses, values greater than 366 min were treated as implausible for a single school-day referral event and set to missing, as described in the Data Analysis section.

#### 2.3.4. Reason for Referral

When a student entered the office, the observer asked why the student had been referred and recorded the response. If the student declined or was unable to provide a reason, the observer obtained the information later from the referring teacher or another school staff member. At the end of the school day, the observer reviewed the recorded reason with the referring teacher and recorded any different or additional information. Thus, a final referral description could incorporate information from more than one informant; separate source-specific reason codes were not retained. The final description was coded into one of six mutually exclusive categories: insubordination, physical aggression, verbal aggression, disruption, destruction of property, or other.

The original coding labels were operationalized as follows: insubordination indicated refusal or failure to comply with an adult direction or school rule; physical aggression indicated fighting or other physical aggression directed toward a peer; verbal aggression indicated verbally aggressive behavior directed toward a peer; disruption indicated excessive talking or other behavior that disrupted the ongoing activity; destruction of property indicated damaging or attempting to damage property; and other included descriptions not covered by the five specified categories, including bus-related behavior.

To assess coding reliability, a second coder independently coded 59 referral events (17.0% of the total sample). The two coders agreed on 86.4% of cases, with Cohen’s κ = 0.817, indicating strong agreement. For analyses focused on school-managed aggression, physical aggression, verbal aggression, and destruction of property were combined into an aggressive/destructive referral category; all remaining referral reasons were classified as other referral reasons. The reported agreement evaluates independent coding of the recorded referral descriptions, not concurrent observation of referral events. Because one research assistant covered each school, duplicate-observer data were not collected for office entry and exit times. Teacher strategies and pre-referral time were teacher reports rather than observer-coded measures.

#### 2.3.5. Teacher Strategies

At the end of the school day, referring teachers were asked an open-ended question about what they had tried before sending the student to the office. Responses were coded into the following categories: verbal warning or prompt/redirect, change of seat, time out, loss of privilege, physical intervention, contact with home, or no strategy reported. Teachers could report more than one strategy for a referral.

#### 2.3.6. Teacher-Reported Time Before Referral

Teachers were also asked to estimate how many minutes they had spent attempting to manage the student’s behavior before referral. For analysis, these reports were also dichotomized to distinguish immediate referrals, for which teachers reported no time spent managing the behavior before referral, from referrals that followed some period of classroom management.

### 2.4. Data Analysis

Data were analyzed using R version 4.6.1. Analyses were primarily descriptive and were conducted at the referral-event level unless otherwise specified. Referral rates were calculated as the number of observed referral events per 100 enrolled students per week. Student-level referral percentages were calculated as the number of unique students referred at least once divided by the number of enrolled students in each condition. Teacher-strategy percentages were calculated as the proportion of referrals with available teacher-strategy information for which each strategy was reported. Because teachers could report more than one strategy for a referral, these percentages could sum to more than 100%. For time-in-office analyses, five referrals with total-time values greater than 366 min were treated as implausible for a single school-day referral event and set to missing. Students were identified by the combination of school and student code. The number of referrals per student was summarized to characterize repeated referral events during the observation week.

To examine whether time spent in the office differed between aggressive/destructive and other referrals while accounting for clustering of referrals within schools, linear regression models were estimated with school fixed effects and CR2 cluster-robust standard errors clustered by school. Small-sample inference used Satterthwaite-adjusted degrees of freedom (Pustejovsky & Tipton, 2018). Because time-in-office values were positively skewed, the analysis was repeated using log-transformed time, log(1 + min), as a sensitivity analysis. Clustering at the school level permits arbitrary dependence among referrals within a school, including multiple referrals involving the same student. As an additional sensitivity analysis, the time-in-office models were repeated after retaining only each student’s first observed referral during the study week.

Because intervention assignment occurred at the school level within seven matched pairs, exploratory intervention–comparison analyses preserved the original matched-pair randomization (Bai et al., 2024; Imai et al., 2009). Overall differences in the six-category referral-reason distribution and five-category time-in-office distribution were evaluated using exact matched-pair randomization tests. Intervention and comparison labels were permuted within each matched pair across all 2^7^ = 128 possible assignments, with the Pearson chi-square statistic used to evaluate differences in the resulting pooled distributions.

For focused condition contrasts, effect estimates were calculated at the matched-pair level so that each of the seven randomized school pairs contributed equally. Within each pair, the comparison-school value was subtracted from the intervention-school value, and the seven within-pair differences were averaged. Effects were expressed in their natural units: referrals per 100 enrolled students for aggressive/destructive referral rates and percentage-point differences for the proportion of referrals involving aggressive/destructive behavior, the proportion of referrals resulting in more than 60 min in the office, and the proportion involving immediate referral. Positive values indicate higher values in intervention schools. Ninety-five percent confidence intervals were calculated from the seven paired differences using the t distribution with 6 degrees of freedom. Exact two-sided randomization *p*-values were obtained by reversing the sign of the within-pair difference under all 128 possible intervention–comparison assignments. Because only seven matched pairs were available, these analyses had limited precision; therefore, effect estimates and confidence intervals were emphasized rather than interpreting nonsignificant tests as evidence of equivalence. Given the post-test-only design, single-week observation period, and small number of randomized school pairs, all intervention–comparison analyses were considered exploratory and were not interpreted as definitive tests of intervention efficacy.

### 2.5. Use of Generative Artificial Intelligence

OpenAI ChatGPT (GPT-5.6 Pro; accessed August 2026) was used during manuscript preparation to assist with organization, language revision, reference-format review, and document formatting. The tool did not have independent access to the raw identifiable data and did not make final analytic or interpretive decisions. All numerical results were generated from the study data using R and were reviewed by the authors. The authors reviewed and edited all AI-assisted output and take full responsibility for the manuscript.

## 3. Results

### 3.1. Overview of Observed Office Discipline Referrals

**Figure 1:**
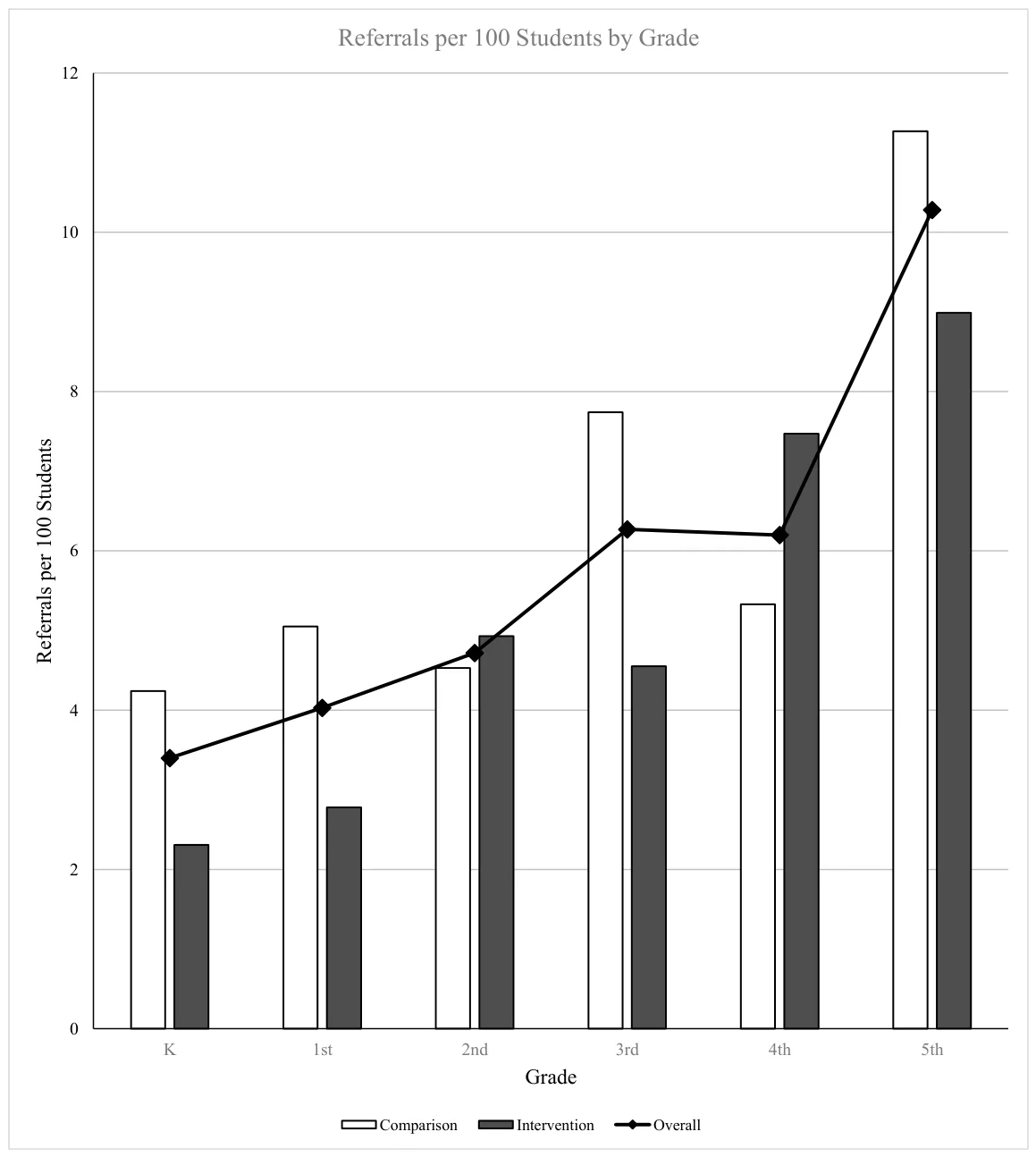
Observed Office Discipline Referrals per 100 Students by Grade.

School characteristics and condition-level referral data are summarized in Table 1. Over the observation period, observers recorded 348 office discipline referral events involving 296 unique students across 14 elementary schools. Referral counts varied substantially across schools, ranging from 4 to 56 events. Comparison schools recorded 212 referral events among 3340 enrolled students, whereas intervention schools recorded 136 referral events among 2670 enrolled students. Thus, observed referral event rates were 6.3 referrals per 100 students per week in comparison schools and 5.1 referrals per 100 students per week in intervention schools. At the student level, 181 students in comparison schools and 115 students in intervention schools were referred at least once during the observation period. Referral event rates generally increased with grade, as shown in Figure 1. Fifth-grade students had the highest observed referral event rate, approximately three times that of kindergarten students. Most referred students (256 of 296; 86.5%) had one referral. Thirty-one students (10.5%) had two referrals, eight (2.7%) had three referrals, and one (0.3%) had six referrals. Thus, 40 students (13.5%) had multiple referrals. The proportion with multiple referrals was similar in intervention schools (17 of 115; 14.8%) and comparison schools (23 of 181; 12.7%).

### 3.2. Reasons for Office Discipline Referral

Table 2 presents referral reasons for the overall sample and by condition. Across all observed referrals, the most common reason was physical aggression, accounting for 134 of 348 referrals (38.5%). The next most common reasons were disruption (98 referrals, 28.2%), insubordination (60 referrals, 17.2%), verbal aggression (19 referrals, 5.5%), destruction of property (10 referrals, 2.9%), and other reasons (27 referrals, 7.8%). The other category included referrals coded as other and referrals for bus behavior.

### 3.3. Aggressive and Destructive Referrals

To examine the aggression-focused pattern more directly, physical aggression, verbal aggression, and property destruction were combined into an aggressive/destructive referral category. Overall, 163 of 348 referrals (46.8%) involved aggressive or destructive behavior. The proportion of referrals involving aggressive/destructive behavior varied by grade. Aggressive/destructive behavior accounted for 61.8% of kindergarten referrals, 52.4% of first-grade referrals, 48.0% of second-grade referrals, 53.3% of third-grade referrals, 54.2% of fourth-grade referrals, and 31.1% of fifth-grade referrals. Thus, although fifth grade had the highest overall referral event rate, fifth-grade referrals were proportionally less likely than those in earlier grades to involve aggressive/destructive behavior.

### 3.4. Time in Office Following Referral

Table 3 summarizes time spent in the office by referral reason and referral type. Among referrals with valid time data, 112 referrals (33.0%) involved 1–15 min in the office; 87 referrals (25.7%) involved 16–30 min; 35 referrals (10.3%) involved 31–45 min; 26 referrals (7.7%) involved 46–60 min; and 79 referrals (23.3%) involved more than 60 min. Thus, nearly one-quarter of referrals with valid time data were associated with more than 1 h in the office following referral.

Aggressive/destructive referrals had longer office stays descriptively than other referrals (*M* = 62.9, SD = 79.4, median = 30 min vs. *M* = 45.8, SD = 67.0, median = 22 min). However, after accounting for school-level clustering and school fixed effects, the estimated within-school difference was 6.4 min and was not statistically significant, b = 6.41, 95% CI [−10.9, 23.8], *p* = 0.425. A sensitivity analysis using log-transformed office time produced the same conclusion, b = 0.196, 95% CI [−0.155, 0.547], *p* = 0.238.

Restricting the analysis to each student’s first observed referral also produced the same substantive conclusion. Among 288 students with valid time data, the estimated within-school difference was 10.8 min, 95% CI [−5.4, 27.0], *p* = 0.166. The corresponding log-transformed model also remained nonsignificant, b = 0.233, 95% CI [−0.099, 0.564], *p* = 0.147.

### 3.5. Teacher-Reported Strategies Before Referral

Teacher-reported pre-referral strategy information was available for 229 referrals (112 comparison and 117 intervention). Because teachers could report more than one strategy for a referral, percentages represent the proportion of referrals with available strategy data in which each strategy was reported. As shown in Table 4, teachers explicitly reported using no classroom strategy before 49.3% of referrals. Verbal warning or prompting was reported for 35.4% of referrals. Seat changes were reported more often in comparison than intervention schools (21.4% vs. 9.4%), whereas loss of privilege was reported more often in intervention than comparison schools (22.2% vs. 10.7%).

Availability of teacher-strategy information differed by condition and school. Strategy information was available for 86.0% of intervention-school referrals (117 of 136) versus 52.8% of comparison-school referrals (112 of 212); school-specific availability ranged from 30.0% to 100%. Availability was relatively similar across grades (61.8–69.0%) but was higher for aggressive/destructive referrals than for other referrals (71.8% vs. 60.5%). Within intervention schools, availability was similar for aggressive/destructive and other referrals (85.1% vs. 87.1%); within comparison schools, the corresponding values were 60.7% and 47.2%. For comparison-school referrals, office-time distributions were similar when strategy information was available versus missing (M = 41.4 vs. 39.4 min; medians = 21 vs. 25 min). In intervention schools, referrals with available strategy information were longer descriptively (M = 77.3 vs. 56.7 min; medians = 30 vs. 24 min). Given the differential availability by condition and school, the strategy comparisons in Table 4 should be interpreted as descriptive available-case results and may be affected by selective reporting.

### 3.6. Exploratory Condition Comparisons

Exploratory intervention–comparison analyses preserved the original matched-pair school-level assignment. The six-category distribution of referral reasons differed descriptively across conditions: intervention schools had proportionally more referrals for physical aggression, verbal aggression, and property destruction and fewer referrals for disruption and other reasons than comparison schools. For example, physical aggression accounted for 41.2% of intervention-school referrals and 36.8% of comparison-school referrals, whereas disruption accounted for 22.1% and 32.1%, respectively. However, the overall referral-reason distribution did not differ significantly between conditions in the exact matched-pair randomization test (*p* = 0.547). Focused condition contrasts for aggressive/destructive referral rates, referral composition, prolonged office stays, and immediate referrals are summarized in Table 5 using pair-aware estimates that give each of the seven randomized school pairs equal weight.

As shown in Table 5, aggressive/destructive referral event rates were nearly identical when referrals were pooled across conditions, and the pair-aware estimate was also close to zero, providing little evidence of a condition difference in the rate of aggressive/destructive referrals.

In contrast, aggressive/destructive behavior made up a larger share of referrals in intervention schools than in comparison schools. The pair-aware estimate remained appreciable at 11.7 percentage points, but the confidence interval was wide and included both little difference and substantially higher proportions in intervention schools. Thus, the behavioral composition of referrals may have differed by condition, but the magnitude and direction of that difference were estimated imprecisely.

A similar pattern emerged for prolonged office stays. More than 60 min in the office was descriptively more common in intervention schools, and the pair-aware estimate was 12.2 percentage points. However, the confidence interval was again wide, indicating substantial uncertainty. The overall five-category time-in-office distribution also did not differ significantly between conditions in the exact matched-pair randomization test (*p* = 0.313).

Finally, the pooled percentages suggested substantially more immediate referrals in intervention schools, but this contrast was greatly attenuated when the seven matched school pairs were given equal weight. The pair-aware estimate was only 4.3 percentage points, providing little evidence of a consistent condition difference in immediate referral across school pairs.

## 4. Discussion

This study examined office discipline referrals (ODRs) as school-response events that reveal how aggressive and other disruptive behaviors are identified, formalized, and managed by adults in elementary schools. Rather than treating ODRs as simple administrative counts of student misconduct, the study used direct observation of office referral events to describe the behaviors that precipitated referral, the time students spent in the office, and teacher-reported strategies used before referral. Three findings are especially noteworthy. First, nearly half of observed referrals involved school-managed aggression, defined as physical aggression, verbal aggression, or property destruction, with physical aggression alone accounting for more than one-third of all referrals. Second, aggressive/destructive referrals were associated descriptively with longer office stays than other referrals, although the within-school difference was smaller and imprecisely estimated after accounting for school-level clustering. Third, referral patterns varied substantially by grade: fifth-grade students had the highest overall referral rate, whereas kindergarten referrals were proportionally most likely to involve aggressive or destructive behavior. Together, these findings illustrate the value of examining not only whether office referrals occur, but also what behaviors precipitate them and what happens after students enter the disciplinary process.

### 4.1. School-Managed Aggression as a Consequential Subset of Childhood Aggression

One contribution of this study is its direct characterization of aggressive and destructive behavior that resulted in administrative referral and of the school response that followed. Nearly half of observed office referrals involved physical aggression, verbal aggression, or property destruction, with physical aggression alone accounting for the largest share of referral events. This pattern aligns with prior elementary-school referral research that shows physical aggression can be a prominent referral reason, especially in the early elementary grades (Rusby et al., 2007). This finding matters because it shows that elementary-school ODRs were not driven solely by low-level disruption, noncompliance, or classroom rule violations. A substantial portion involved behaviors with immediate implications for peer safety, teacher response, classroom order, and student access to instruction.

At the same time, these data should not be interpreted as prevalence estimates of aggression in school. They reflect aggression that crossed an adult referral threshold. This distinction is important because many studies assess aggression through parent and teacher ratings, self-reports, peer nominations, diagnostic interviews, or structured observations. ODRs capture a different phenomenon: aggressive behavior that has become institutionally consequential. That makes ODRs both limited and valuable. They are limited because they miss aggression that goes unnoticed, is handled informally, or is interpreted differently by adults. They are valuable because they identify episodes of aggression that activate school disciplinary systems and may shape children’s educational experiences. In particular, the referral event rate reported here is a rate of formal office referrals per enrolled student, not a measure of how often aggressive or disruptive behavior occurred. ODRs also do not provide a standardized measure of behavioral intensity and do not capture behavior that was unnoticed or managed without referral.

### 4.2. Instructional Consequences of Aggression-Related Referrals

Aggressive/destructive referrals were associated descriptively with longer office stays than other referrals. Students referred for physical aggression, verbal aggression, or property destruction spent an average of 62.9 min in the office, compared with 45.8 min for other referrals, and the corresponding medians were 30 and 22 min. However, after accounting for differences between schools and clustering of referrals within schools, the estimated within-school difference was considerably smaller, 6.4 min, and imprecisely estimated (95% CI [−10.9, 23.8]). The sensitivity analysis using log-transformed office time led to the same conclusion. Thus, the present data do not establish that aggressive/destructive referrals reliably result in longer office stays, although they remain compatible with a potentially meaningful increase in time outside instruction.

More broadly, the findings demonstrate why referral counts alone provide an incomplete picture of the educational consequences of school discipline. Nearly one-quarter of referrals with valid time data involved more than 60 min in the office. A single referral can therefore represent very different experiences. One student may spend only a few minutes in the office while an administrator helps resolve a conflict and then return to class, whereas another may spend more than an hour outside instruction, missing academic content, peer interaction, and classroom routines. Both events are counted as one ODR.

For research on aggression and school intervention, this distinction is important. Referral counts indicate whether the disciplinary system was activated, but they do not capture the duration or intensity of the resulting removal from instruction. Measuring time in the office therefore provides information about disciplinary consequences that is not available from referral frequency alone. Future research with larger numbers of schools and repeated observations could determine more precisely whether aggressive or destructive incidents systematically produce longer removals than other forms of misconduct.

This point also has practical implications. Schools that rely solely on ODR counts may overlook students, classrooms, or grade levels associated with substantial losses of instructional time. Adding simple fields to referral systems—such as time sent, time returned, reason for referral, whether immediate safety concerns were present, pre-referral strategies attempted, and reentry procedures—could make ODR data more informative for intervention planning and for monitoring the educational consequences of disciplinary responses.

### 4.3. ODRs as Filtered Measures of Aggression

The findings underscore the importance of interpreting ODRs as “filtered” measures of aggression rather than as direct measures of aggressive behavior. That is, they have been filtered through a sequence of events: behavior occurs, an adult observes and interprets it, determines whether it can be managed in the classroom, and decides whether it warrants formal administrative involvement. ODRs therefore reflect both student behavior and adult response processes.

This distinction is particularly important because aggressive and disruptive behaviors differ in how directly they signal danger or the need for removal. Physical aggression and property destruction may be relatively concrete and more likely to prompt immediate administrative involvement, whereas verbal aggression, disruption, insubordination, or disrespect may depend more heavily on adult interpretation and contextual factors. Classroom norms, teacher tolerance, prior student reputation, the student–teacher relationship, school discipline culture, and perceived safety risk may all influence whether a given incident crosses the referral threshold. In this sense, similar behavioral disturbances can produce different consequences under different surrounding conditions, as occurs in some natural systems (Mack & Hebert, 1997, 1999).

Consequently, differences in ODRs cannot be attributed automatically to differences in students’ aggressive behavior. They may also reflect variation in adult labeling, classroom-management practices, referral thresholds, or administrative procedures (Girvan et al., 2017; Martella et al., 2010; Wright & Dusek, 1998). This does not make ODRs uninformative. Rather, it clarifies what they measure: behavior that has been observed, interpreted, and judged sufficiently consequential to activate the school disciplinary system. For aggression research, that is a meaningful outcome, but one that should be distinguished from the underlying prevalence of aggressive behavior.

### 4.4. Developmental and Grade-Related Patterns

The grade-related findings provide useful developmental context for interpreting school-managed aggression. Fifth-grade students had the highest overall referral rate, whereas kindergarten students had the highest proportion of referrals involving physical aggression, verbal aggression, or property destruction. Although these data are cross-sectional and based on a single observation week, the pattern is broadly consistent with prior referral research suggesting that aggressive referral reasons may be especially prominent among younger elementary-school students, whereas referrals among older students may reflect a broader mix of disruption, noncompliance, disrespect, and other rule violations (Kaufman et al., 2010; Rusby et al., 2007).

These differences may have implications for how schools interpret and respond to ODR data across grade levels. In earlier grades, a greater concentration of aggression-related referrals may point to the importance of developmentally appropriate supports for emotion regulation, peer conflict, safe social interaction, and adult-guided de-escalation. In later elementary grades, a broader mix of referral reasons may place relatively greater emphasis on classroom-management systems, behavioral expectations, academic engagement, and patterns of interaction between students and adults. These interpretations remain hypotheses for future research rather than conclusions that can be established from the present cross-sectional data.

More generally, the grade pattern cautions against treating ODRs as developmentally uniform indicators of behavior problems. The meaning of a referral may differ depending on a student’s age, the form of behavior involved, classroom expectations, and the context in which the incident occurs. ODR data may therefore be most informative when behavioral form and developmental level are considered together rather than when referral counts are interpreted in isolation.

### 4.5. Teacher-Reported Pre-Referral Responses

Teacher-reported classroom strategy data provide additional insight into the classroom-management processes that preceded office referral. Among referrals with available strategy information, teachers explicitly reported no classroom strategy before nearly half of referrals, whereas a verbal warning or prompt was reported for approximately one-third. These findings suggest that many referrals were reported as occurring without a preceding classroom-management strategy or after relatively limited intervention. However, they should not be interpreted to mean that teachers necessarily did nothing before referral. Strategy information was obtained retrospectively, was unavailable for approximately one-third of referrals, and may not have captured informal or routine responses that teachers did not recall or report. Availability was higher in intervention than comparison schools and varied widely across individual schools. Consequently, the observed condition differences in specific strategies may partly reflect which referrals had teacher follow-up data rather than underlying differences in classroom practice.

Immediate referral may also have been appropriate for some incidents. Physical aggression, threats, property destruction, or other behaviors involving immediate safety concerns may reasonably require administrative involvement without an extended sequence of classroom interventions. In contrast, some disruptive or non-dangerous behaviors may be manageable through brief classroom-based strategies such as redirection, reinforcement, structured choices, or de-escalation (Fabiano et al., 2024; Waschbusch et al., 2019). Thus, the number or type of strategies attempted before referral cannot be interpreted independently of the behavior that precipitated the referral.

Nevertheless, the period immediately preceding referral represents a potentially important intervention point. ODRs reflect not only student behavior but also decisions about when classroom management has been exhausted or when immediate removal is necessary. Future studies that directly observe both the behavior preceding referral and teachers’ responses to it could help distinguish appropriate immediate referrals from incidents that might be safely managed within the classroom. Such information could inform clearer decision rules for responding to aggression while reducing unnecessary removal from instruction.

### 4.6. Exploratory Intervention-Condition Differences

Exploratory comparisons between intervention and comparison schools did not provide clear evidence of condition differences once the original matched-pair school-level assignment was taken into account. Aggressive/destructive referral rates were nearly identical when referrals were pooled across conditions, and the pair-aware estimate was also close to zero. Thus, the data provide little indication that intervention schools differed from comparison schools in the overall rate at which aggressive or destructive behavior resulted in office referral.

The behavioral composition of referrals showed a somewhat different pattern. Aggressive/destructive behavior accounted for 54.4% of referrals in intervention schools compared with 42.0% in comparison schools, and the pair-aware estimate remained appreciable at 11.7 percentage points. Similarly, office stays longer than 60 min were more common (at least descriptively) in intervention schools, with a pair-aware difference of 12.2 percentage points. However, confidence intervals for both estimates were wide and included substantial differences in either direction. These findings therefore do not establish condition effects, but they also do not rule out potentially meaningful differences in referral composition or prolonged office removal.

The immediate-referral finding was less consistent across school pairs. Although pooled percentages suggested a large difference between conditions, the pair-aware estimate was only 4.3 percentage points. This indicates that the pooled contrast was influenced by the unequal number of referral observations contributed by individual schools and was not representative of a consistent intervention–comparison difference across the seven randomized pairs.

Taken together, these analyses argue against interpreting the observed condition differences as evidence that the ABC intervention altered student behavior or school referral practices. At the same time, the limited number of randomized school pairs resulted in substantial uncertainty for some outcomes. The present findings are therefore more informative about the range of effects compatible with the data than about whether an intervention effect was definitively present or absent.

Even so, the results are consistent with the possibility, which could be addressed in a future study, that school-wide behavioral interventions may alter not only the frequency of referrals but also their composition. For example, improved classroom management of lower-intensity disruption or noncompliance could leave a referral pool more concentrated on aggression, safety concerns, or other behaviors requiring administrative involvement. Such a mechanism would be broadly consistent with features of the ABC intervention (Pelham et al., 2005; Waschbusch et al., 2005), but the present post-test-only data cannot test it. Repeated observations before and after intervention in a larger number of randomized schools would be needed to distinguish intervention-related changes from preexisting differences, school discipline culture, referral thresholds, administrative practices, or chance variation.

More broadly, prior research suggests that school-wide prevention and intervention programs can affect ODR patterns (Bocquillon et al., 2025; Sugai et al., 2000). Future intervention studies should therefore examine not only referral frequency but also the behavioral composition of referrals, pre-referral classroom strategies, duration of office removal, and reentry procedures. These outcomes may reveal changes in how schools manage aggressive and disruptive behavior that would be missed by referral counts alone.

### 4.7. Implications for Research and School Practice

The findings have implications for both aggression research and school-based practice. For researchers, ODRs may be most informative when conceptualized as indicators of school-managed behavior rather than as direct measures of aggression prevalence. This is because they identify incidents that have passed through adult observation and decision-making and have become sufficiently consequential to activate a formal school response. This makes ODRs particularly useful for studying the intersection of child behavior and school response, but it also means that referral data should be interpreted in conjunction with other measures when the goal is to characterize children’s broader aggressive behavior.

Aggression research may also benefit from examining what happens after an aggressive incident occurs; this may be an important and interesting outcome in its own right. Teacher and administrator responses are part of a behavioral context in which aggression occurs, and observational research has emphasized the importance of examining reciprocal sequences between child behavior and adult responses (Vujnovic et al., 2014). In the context of ODRs, potentially informative outcomes include whether a student is removed from instruction, how long that removal lasts, what occurs in the office, and how the student returns to the classroom. Combining these measures with teacher ratings, direct observation, universal screening, and longitudinal student data could help distinguish aggressive behavior itself from the school processes that follow it.

For schools, the results suggest that the usefulness of ODR data depends partly on the information collected with each referral. Referral counts alone cannot distinguish a brief administrative contact from an extended removal from instruction, nor can they show what was attempted before referral or how the student was supported afterward. Recording a small number of additional process indicators could allow school teams to examine not only how frequently referrals occur but also which behaviors produce referrals and what educational consequences follow them. Developing a standardized ODR measurement framework could make these data more comparable and more useful. At minimum, such a framework would distinguish (a) classroom and student context, including class size and composition, grade, student disability or special-education status, and relevant school or district policy; (b) the precipitating behavior, using operational definitions and indicators of severity or intensity; (c) pre-referral responses, including classroom strategies and teacher time; (d) the office response, including staff involved, duration, counseling or therapeutic support, parent or caregiver contact, and disciplinary consequences; and (e) reentry and follow-up. Common definitions across these stages would support comparison across schools and studies without reducing ODRs to a single event count.

More broadly, intervention can occur at multiple points in the referral sequence (Waschbusch et al., 2019). Before an incident, schools can strengthen classroom management, behavioral expectations, supervision, and supports for emotion regulation and peer conflict. During an incident, staff need clear guidance for distinguishing situations requiring immediate removal for safety from those that can be managed effectively in the classroom. After referral, efficient administrative procedures and planned return to instruction may help limit unnecessary exclusion and support successful reentry. The goal should therefore not be simply to minimize the number of ODRs. Some aggressive incidents warrant immediate formal intervention. A more useful goal is to ensure that referrals occur when needed, preserve safety, minimize unnecessary loss of instruction, and lead to an appropriate response to the behavior that prompted them. Implementing this approach also requires teacher preparation in developmentally appropriate classroom management, de-escalation, and discipline, together with clear coordination among teachers, administrators, caregivers, and school-based mental-health personnel.

### 4.8. Limitations

Several limitations should be noted. First, the study used a post-test-only design and included only seven matched pairs of schools. Although schools were randomly assigned to condition within pairs, comparable pre-intervention observational data were unavailable. The small number of randomized pairs substantially limited the precision of condition-effect estimates. For some outcomes, such as the proportion of referrals involving aggressive/destructive behavior and the proportion involving more than 60 min in the office, the pair-aware point estimates were potentially meaningful but the confidence intervals were wide and compatible with both little difference and considerably larger condition differences. Thus, nonsignificant results for these outcomes should not be interpreted as evidence of equivalence. At the same time, limited precision does not explain all null findings: the pair-aware aggressive/destructive referral-rate difference was close to zero, and the pooled difference in immediate referral was substantially attenuated when the seven matched pairs were weighted equally. Accordingly, the condition comparisons are best interpreted as exploratory estimates rather than definitive evidence for or against intervention effects.

Second, referral events were observed at each school during only one designated week between early May and early June, near the end of the school year. Intensive observation made it possible to characterize individual referral events in considerably greater detail than was available from administrative records, but a one-week sample from each school may not represent disciplinary patterns across the full school year. Referral frequency and composition may vary with time of year, classroom context, school events, testing periods, teacher and student familiarity with routines, and other situational factors. Repeated observations across the school year would provide a stronger basis for estimating typical referral patterns and distinguishing stable school differences from short-term variation.

Third, the observations were conducted in 2007 as part of the broader SACD study. School discipline policies, behavioral-support practices, documentation systems, and broader educational contexts may have changed since these data were collected. The detailed referral-process data remain useful for examining how aggressive behavior becomes formalized and managed within school discipline systems, but the specific referral frequencies, response patterns, and durations observed here should not be assumed to characterize contemporary elementary schools. Replication using current school discipline practices is needed.

Fourth, observation began after students entered the administrative office and therefore did not capture the full classroom sequence preceding referral. Teacher-reported strategy data provided information about what teachers recalled doing before referral, but these reports were retrospective and their validity could not be independently established. Moreover, strategy information was unavailable for 119 referrals (34.2%). Availability differed markedly by condition and school, and some differences were also observed by referral category. Consequently, available-case strategy estimates may be affected by selective reporting, and the descriptive condition comparisons should not be interpreted as causal intervention effects. The study also could not determine how often aggressive or disruptive behavior occurred without resulting in referral, how such incidents were handled in classrooms, or why some behaviors crossed the referral threshold whereas others did not.

Fifth, reliability was assessed only for the coding of referral reasons. The study did not include concurrent second observers for office entry and exit times, and the accuracy of those timestamps therefore could not be independently verified. The teacher-strategy and pre-referral-time measures were retrospective teacher reports rather than direct observations, and their reliability was not assessed. Findings involving time in the office and teacher-reported measures should therefore be interpreted as descriptive estimates from this observation protocol.

Sixth, referral reasons were coded from descriptions of observed disciplinary events rather than from standardized behavioral assessments. Although interrater reliability was strong, categories such as physical aggression, verbal aggression, disruption, and insubordination are school-discipline classifications rather than comprehensive measures of child behavior. The aggressive/destructive grouping was useful for identifying externally directed behaviors with potential safety or disciplinary significance, but it combined behaviors that may differ in severity, function, intent, and developmental meaning. The study also did not measure important features of aggressive incidents such as provocation, reactive versus proactive function, injury or threat severity, or the identity of the target.

Finally, several potentially important student-, teacher-, classroom-, administrator-, school-, and district-level variables were unavailable. In particular, school and district discipline policies were not measured in a form that permitted direct analysis. Random assignment within matched school pairs protects the exploratory condition contrasts in expectation, and school fixed effects account for stable between-school differences in the within-school time analysis, but neither feature eliminates unmeasured heterogeneity in discipline policy, referral thresholds, or administrative practice. The analyses also could not account for class size, classroom composition, students’ prior disciplinary histories, diagnostic or disability status, academic functioning, teacher characteristics or discipline training, classroom-management quality, administrator decision-making, or the specific interventions and consequences delivered in the office. Such variables may influence both whether behavior results in referral and what occurs afterward (Bradshaw et al., 2010; Girvan et al., 2017; Rocque, 2010). Future studies that integrate these contextual variables with direct observation could more clearly separate student behavior, adult response, and school-level disciplinary processes.

### 4.9. Future Directions

Future research should examine ODRs as part of a broader behavioral and disciplinary process rather than as isolated administrative outcomes. Ideally, studies would begin with direct observation of the classroom event, follow the teacher’s response and referral decision, document what occurs in the office, and examine the student’s return to instruction. Integrating these observations with teacher and student reports, administrative records, and measures of student functioning would help distinguish the contributions of student behavior, adult decision-making, and school disciplinary practices.

Longitudinal and repeated-observation designs are also needed. Sampling referral events at multiple points across the school year would clarify the stability of aggressive/destructive referral patterns and office-removal duration, while following students over time could determine whether repeated or extended referrals predict subsequent academic, behavioral, or disciplinary outcomes. Such studies should also examine student, classroom, teacher, and school characteristics that may influence whether aggressive behavior results in formal referral, including class size and composition, student disability or special-education status, teacher preparation in classroom management and discipline, and school or district referral policy.

Finally, intervention studies should evaluate multiple components of the referral process rather than relying solely on referral counts. A standardized classification of office responses could distinguish brief problem solving or inductive discipline, counseling or school-based mental-health referral, restitution or other restorative responses, parent or caregiver liaison, brief or prolonged suspension, and planned reentry supports. Linking these response categories to operationally defined behavior types and outcomes such as repeated referrals, recurrence of the target behavior, time outside instruction, and successful return to class could test whether particular responses are more effective for particular behavioral problems. Larger cluster-randomized studies with observations before and after intervention could also test whether school-wide or classroom-based programs change the frequency and behavioral composition of referrals, teacher responses before referral, duration of removal from instruction, administrative responses, and reentry practices. This approach would help determine whether interventions affect aggressive behavior itself, adults’ responses to that behavior, or both.

## 5. Conclusions

This study used direct observation of elementary-school office referral events to characterize how aggressive and other disruptive behaviors were identified and managed through school disciplinary procedures. Nearly half of observed referrals involved physical aggression, verbal aggression, or property destruction, underscoring the importance of ODRs as indicators of school-managed aggression. Aggressive/destructive referrals involved descriptively longer office stays than other referrals, although the within-school difference was smaller and imprecisely estimated after accounting for school-level clustering. Referral patterns also varied by grade, with fifth-grade students showing the highest overall referral rate and kindergarten referrals proportionally most likely to involve aggressive/destructive behavior.

More broadly, ODRs should not be interpreted as simple measures of aggression prevalence. They reflect both student behavior and the sequence of adult observations and decisions that determine whether behavior becomes a formal disciplinary event. Understanding aggression in schools therefore requires attention not only to whether referrals occur, but also to what precipitates them, how adults respond, how much instruction students miss, and how students return to the classroom. Examining these processes may make ODR data more useful for both aggression research and school-based intervention.

## Figures and Tables

**Table 1:** School Characteristics and Office Discipline Referral Data by Condition.

Variable	Overall	Comparison	Intervention
Sample Size			
Number of Schools	14	7	7
Number of Students	6010	3340	2670
Student Demographics, Mean % (SD)			
Male	50.36 (3.31)	50.73 (2.83)	49.99 (3.93)
Black	50.19 (33.31)	50.11 (33.50)	50.28 (35.74)
White	33.17 (32.29)	33.87 (31.20)	32.46 (35.82)
Hispanic	14.97 (21.85)	14.65 (21.90)	15.29 (23.46)
Free Lunch status	66.67 (26.44)	69.41 (26.50)	63.92 (28.11)
Reduced Lunch status	13.82 (12.17)	10.91 (6.95)	16.72 (15.91)
Total Office Referrals			
Observed referrals, *n*	348	212	136
Students referred at least once, *n* (%)	296 (4.9%)	181 (5.4%)	115 (4.3%)
Referrals per 100 students	5.8	6.3	5.1
Aggressive/Destructive Referrals			
Observed referrals, *n*	163	89	74
Referrals per 100 students	2.7	2.7	2.8
Percentage of referrals aggressive/destructive	46.8%	42.0%	54.4%

Note: Demographic values are school-level percentages averaged across schools. Referrals per 100 students are event rates, calculated as the number of observed referrals per 100 enrolled students. Percentages for students referred at least once are calculated as the number of unique referred students divided by the number of enrolled students in each condition.

**Table 2:** Reasons for Observed Office Discipline Referrals by Condition.

	Overall Sample		Comparison		Intervention	
Referral Reason	n	%	n	%	n	%
Physical aggression	134	38.5	78	36.8	56	41.2
Disruption	98	28.2	68	32.1	30	22.1
Insubordination	60	17.2	33	15.6	27	19.9
Verbal aggression	19	5.5	8	3.8	11	8.1
Destruction of property	10	2.9	3	1.4	7	5.1
Other	27	7.8	22	10.4	5	3.7

Note: Percentages are column percentages. “Other” includes referrals originally coded as other and bus behavior.

**Table 3:** Minutes in Office Following Office Discipline Referral by Referral Reason and Referral Type.

Referral Reason/Type	n	M (SD)	Median	75th Percentile	>60 min, n (%)
Specific referral reason					
Physical aggression	128	67.6 (84.5)	30.5	88.0	38 (29.7)
Disruption	98	42.7 (55.4)	25.0	48.8	21 (21.4)
Insubordination	59	30.8 (30.1)	22.0	45.0	7 (11.9)
Verbal aggression	17	36.3 (38.8)	20.0	43.0	3 (17.6)
Destruction of property	10	48.1 (48.4)	30.0	41.2	2 (20.0)
Other	27	89.8 (124.7)	20.0	155.0	8 (29.6)
Collapsed referral type					
Aggressive/destructive	155	62.9 (79.4)	30.0	75.0	43 (27.7)
Other referral reason	184	45.8 (67.0)	22.0	50.0	36 (19.6)

Note: Values are minutes spent in the office following an office discipline referral, calculated from observed office entry and exit times. Four referrals were missing total-time values. Five additional referrals with total-time values greater than 366 min were treated as implausible for a single school-day referral event and excluded from these analyses. Aggressive/destructive referrals include physical aggression, verbal aggression, and destruction of property. The collapsed referral-type rows are summaries of the specific referral-reason rows, not additional categories.

**Table 4:** Teacher-Reported Strategies Used Before Office Discipline Referral by Condition.

Strategy	Overall n (%)	Comparison n (%)	Intervention n (%)
No strategy reported	113 (49.3%)	50 (44.6%)	63 (53.8%)
Verbal warning/prompt	81 (35.4%)	39 (34.8%)	42 (35.9%)
Seat change	35 (15.3%)	24 (21.4%)	11 (9.4%)
Time out	21 (9.2%)	9 (8.0%)	12 (10.3%)
Loss of privilege	38 (16.6%)	12 (10.7%)	26 (22.2%)
Contacted home	16 (7.0%)	6 (5.4%)	10 (8.5%)
Physical intervention	5 (2.2%)	3 (2.7%)	2 (1.7%)

Note: Teacher-strategy information was available for 229 of 348 referral events (112 comparison and 117 intervention). Percentages represent the proportion of referrals with available teacher-strategy data for which each strategy was reported. Teachers could report more than one strategy for a referral; therefore, percentages sum to more than 100%. Verbal warning and prompting/redirect were combined into one category. “No strategy reported” indicates that the teacher explicitly reported using no classroom strategy before the referral.

**Table 5:** Exploratory Matched-Pair Estimates of Intervention–Comparison Differences.

Outcome	Comparison	Intervention	Pair-Aware Estimate (I − C)	95% CI	Exact *p*
Aggressive/destructive referrals per 100 students	2.7	2.8	−0.37	[−3.27, 2.52]	0.797
Aggressive/destructive referrals, %	42.0	54.4	11.7 pp	[−10.4, 33.7]	0.250
Office stays >60 min, %	16.9	33.3	12.2 pp	[−16.0, 40.4]	0.344
Immediate referrals, %	27.8	47.7	4.3 pp	[−25.0, 33.6]	0.688

Note: Comparison and intervention values are pooled descriptive values. Pair-aware estimates are the mean of the seven within-pair intervention-minus-comparison differences, giving each randomized school pair equal weight. Confidence intervals are paired-*t* intervals based on the seven pair differences. Exact two-sided *p* values were obtained from all 128 possible within-pair intervention–comparison assignments. pp = percentage points.

## Data Availability

The data presented in this study are available on request from the corresponding author. Public availability is restricted by institutional and participating school-district requirements designed to protect participant privacy; requests for access are therefore subject to applicable institutional and school-district approval. Analysis code is available from the corresponding author upon reasonable request.
